# Cognitive Weighting of Constraints on Exercise Participation: A Conjoint Analysis

**DOI:** 10.3390/bs16060976

**Published:** 2026-06-12

**Authors:** Won-Yong Jang, Eui-Yul Choi

**Affiliations:** 1 Urban Housing Research Institution, Gwangju Metropolitan Corporation, Gwangju 61964, Republic of Korea; overm200@nate.com; 2 Department of Leisure Sports Tourism, Youngsan University, Busan 48015, Republic of Korea

**Keywords:** exercise participation, constraints, cognitive weighting, conjoint analysis, segmentation, exercise involvement

## Abstract

This study examines how adults with recent exercise participation experience cognitively weigh different perceived constraints on exercise participation and whether these weighting structures can be used for meaningful segmentation. The sample included 283 adults aged 19 years and older who had engaged in exercise at least once per week for a minimum of 30 min over the past three months. This study aimed to identify the relative importance of perceived exercise participation constraints among adult exercisers, examine differences according to involvement level, and segment participants based on constraint importance. The results showed that, within this sample, intrapersonal constraints, particularly lack of interest and physical fatigue, were the most influential, followed by structural constraints such as time and cost. Constraint prioritization varied by involvement level: highly involved individuals emphasized time burden, whereas less involved individuals highlighted a lack of interest. Cluster analysis identified four distinct segments: interest-constrained beginners, fatigue-sensitive participants, time-constrained active participants, and cost-sensitive experienced participants. These segments differed significantly in demographic and behavioral characteristics, including age, exercise frequency, and participation duration. Overall, the findings suggest that among adults with recent exercise participation experience, perceived exercise participation constraints are cognitively weighted and vary across individuals. This study contributes by applying conjoint analysis to assess the relative importance of multiple perceived constraints and by providing a segmentation-based perspective on how adult exercisers perceive constraints.

## 1. Introduction

Regular participation in physical activity is widely recognized as a critical determinant of physical health, psychological well-being, and overall quality of life. Accumulating evidence has consistently demonstrated that sustained engagement in exercise contributes to the prevention of chronic diseases (Warburton & Bredin, 2017), as well as improvement in mental health and overall life satisfaction (Rhodes et al., 2017). Nevertheless, despite these well-established benefits, a substantial proportion of individuals remain physically inactive or fail to maintain regular participation over time. This persistent gap between knowledge and behavior suggests that the determinants of exercise participation extend beyond awareness and intention, requiring a more nuanced understanding of the decision-making processes underlying exercise participation (Ajzen, 1991; Payne et al., 1993; Wan et al., 2024).

Recent research has increasingly emphasized that exercise participation behavior is shaped by dynamic interactions among psychological, social, environmental, and contextual factors rather than by isolated barriers. Contemporary physical activity research suggests that participation decisions emerge from complex configurations of determinants that vary across individuals and situations (Rhodes et al., 2017; Wan et al., 2024). Furthermore, recent systematic reviews have demonstrated that interventions targeting multiple behavioral determinants simultaneously are generally more effective than those focusing on a single factor alone (Wan et al., 2024). These developments indicate a growing need to move beyond traditional variable-centered approaches and to better understand how individuals prioritize and evaluate multiple exercise constraints when making participation decisions.

Within the domain of leisure and sport research, constraints have long been regarded as key factors influencing participation behavior. Traditional conceptualizations classify constraints into intrapersonal, interpersonal, and structural dimensions, reflecting psychological, social, and environmental barriers, respectively (Crawford & Godbey, 1987; Jackson et al., 1993). While this framework has been instrumental in identifying various barriers to exercise, recent studies have increasingly conceptualized exercise constraints as subjective and context-dependent experiences that influence how individuals perceive, interpret, and evaluate participation opportunities (H. Cho et al., 2024; S.-Y. Kim, 2024; Wan et al., 2024).

Building on this conceptual shift, recent empirical studies have continued to identify time scarcity, financial cost, physical fatigue, and lack of interest as major barriers to exercise participation (S.-Y. Kim, 2024). These findings suggest that exercise participation decisions are rarely determined by a single constraint; rather, they emerge from the combined influence of multiple competing barriers. However, despite recognizing this complexity, most previous studies have relied on analytical approaches that examine individual constraints independently, providing limited insight into how individuals cognitively prioritize and evaluate multiple constraints simultaneously.

From the perspective of leisure constraints theory, individuals often experience multiple intrapersonal, interpersonal, and structural constraints simultaneously (Crawford & Godbey, 1987; Jackson et al., 1993). However, while the theory successfully explains the types of constraints that inhibit participation, it provides a limited explanation regarding how these competing constraints are cognitively prioritized during participation decisions. Multi-attribute decision-making theory offers a complementary perspective by suggesting that individuals assign differential importance to multiple attributes and make decisions based on the resulting trade-offs (Payne et al., 1993). Integrating these perspectives implies that exercise participation decisions should be understood not only in terms of the existence of constraints but also in terms of the relative weight assigned to each constraint within the decision-making process.

Contemporary behavioral theories similarly suggest that behavior emerges from the integration and evaluation of multiple determinants rather than from the influence of a single dominant factor (Ajzen, 1991; Wan et al., 2024). Nevertheless, limited empirical evidence exists regarding the relative importance individuals assign to competing exercise constraints. Addressing this gap requires analytical approaches capable of capturing the psychological weighting and trade-off evaluation processes underlying exercise participation decisions. In this study, cognitive weighting refers to the relative importance that respondents assign to different perceived constraints when evaluating conjoint profiles of exercise participation. It does not imply the direct observation of stable or unconscious cognitive mechanisms. Rather, cognitive weighting is operationally defined as an inferred evaluative structure derived from respondents’ ranking of hypothetical constraint profiles. Accordingly, the part-worth utilities and relative importance values estimated through conjoint analysis are interpreted as indicators of task-based perceived constraint prioritization, not as direct measures of actual cognitive processes.

To address this gap, the present study adopts conjoint analysis, a methodological approach that enables the estimation of the relative importance of multiple attributes within a decision-making context. Conjoint analysis has been widely used in consumer research to examine how individuals make trade-offs among competing factors (Green & Srinivasan, 1990; Orme, 2010). By presenting respondents with hypothetical scenarios that combine different constraint attributes, this method allows for the extraction of implicit importance weights assigned to each constraint (Orme, 2010). In the context of exercise participation, this approach provides a more realistic representation of how individuals evaluate and prioritize multiple barriers simultaneously.

Furthermore, this study incorporates exercise involvement as a key moderating variable. Exercise involvement refers to the perceived personal relevance and importance of exercise, which influences individuals’ motivation, commitment, and persistence (Kyle et al., 2006). Prior research has demonstrated that individuals with higher involvement are more likely to overcome perceived barriers, whereas those with lower involvement are more susceptible to constraints (Havitz & Dimanche, 1997). In addition, recent perspectives emphasize that the effects of physical activity and its associated barriers are not uniform across individuals, underscoring the importance of considering psychological heterogeneity (Wan et al., 2024).

Recent research has further emphasized that the influence of exercise-related barriers is not uniform across individuals and may vary according to their motivational orientation and level of exercise involvement (Wan et al., 2024). This highlights the importance of examining heterogeneity in the perception and weighting of exercise constraints. Despite these insights, limited research has examined how involvement shapes the relative importance assigned to different constraints. It is plausible that highly involved individuals may downplay structural constraints such as time or cost, while low-involvement individuals may perceive these constraints as decisive barriers. Understanding such differences is critical for explaining variability in exercise behavior and for developing targeted interventions. Based on these theoretical considerations, the present study integrates leisure constraints theory, multi-attribute decision-making theory, and exercise involvement theory to examine not only the existence of exercise constraints but also their relative importance in exercise participation decision-making. Specifically, it addresses the following research questions:

1. Among adults with recent exercise participation experience, which perceived constraints exert the strongest psychological inhibitory influence on exercise participation decision-making?
2. Among adult exercisers, how do the relative weightings of these perceived constraints vary between individuals with high and low levels of exercise involvement?
3. How can adult exercisers be segmented based on the relative importance assigned to perceived exercise participation constraints, and how do these segments differ in demographic and exercise-related characteristics?

By addressing these questions, this study contributes to the literature in several important ways. First, it advances prior research by shifting the analytical focus from isolated constraint effects to the psychological weighting and relative importance of constraints within a decision-making framework. Second, it introduces conjoint analysis as a methodological tool capable of capturing the complexity of real-world behavioral choices and the trade-off evaluation processes underlying exercise participation decisions. Third, it incorporates exercise involvement as a moderating factor, thereby providing a more nuanced understanding of individual differences in constraint perception.

Ultimately, this study offers a psychologically grounded framework for understanding exercise participation behavior. By identifying the most influential constraints and examining how their importance varies across individuals, the findings contribute actionable insights for the development of more effective, evidence-based strategies to promote physical activity.

## 2. Materials and Methods

### 2.1. Data Collection

Data collection was conducted through an online survey platform over two months. All respondents were informed of the purpose of the study and assured that their responses would remain anonymous, and voluntary participation was confirmed before data collection began. A total of 300 questionnaires were distributed. After removing 17 incomplete or unusable responses, 283 valid cases were retained for analysis. The sample should be interpreted as representing adult exercisers who satisfied the specified participation criteria rather than the general population. Because recruitment was conducted through an online survey platform, the sample may have included respondents with relatively greater digital accessibility or health-related interest than the broader adult population. The sampling approach intentionally focused on individuals with recent exercise experience to ensure that participants could realistically evaluate the constraints on exercise participation in the conjoint analysis.

The overall survey process consisted of two stages: a preliminary attribute-development stage and a main survey stage. In the preliminary attribute-development stage, an open-ended survey, literature review, and expert consultation were used to identify and refine the constraint attributes and levels for the conjoint analysis. After the attributes and levels were finalized, conjoint profiles were generated using an orthogonal fractional factorial design. The main survey was then conducted to collect demographic and exercise-related characteristics, respondents’ rankings of the conjoint profiles, and exercise involvement scores.

Regarding sample size considerations, prior research indicates that conjoint analysis does not require a rigid minimum number of respondents; however, larger samples yield greater reliability and stability of results. Green and Srinivasan (1990) suggested that at least 100 respondents are desirable for reliable conjoint estimation (as cited in Novotorova (2007)). Based on these methodological considerations, the final sample of 283 respondents was deemed adequate for estimating part-worth utilities and comparing preference weight structures across exercise involvement groups.

A total of 283 valid responses were included in the final analysis. In terms of gender, 57.2% (n = 162) of the respondents were male, while 42.8% (n = 121) were female. Regarding age distribution, 22.6% (n = 64) were in their 20s, 31.4% (n = 89) were in their 30s, 27.6% (n = 78) were in their 40s, and 18.4% (n = 52) were aged 50 years or older. Regarding weekly exercise frequency, 37.8% (n = 107) reported exercising 1–2 times per week, 46.3% (n = 131) exercised 3–4 times per week, and 15.9% (n = 45) exercised every day. In terms of exercise participation duration, 19.1% (n = 54) had been exercising for less than 6 months, 34.6% (n = 98) for 6 months to less than 1 year, 28.3% (n = 80) for 1 to less than 3 years, and 18.0% (n = 51) for 3 years or more. The specific demographic characteristics of the respondents are presented in Table 1.

### 2.2. Measurement

The present study employed conjoint analysis to address three primary research objectives. First, it aimed to evaluate the cognitive weighting of perceived constraints by estimating their relative importance in exercise participation decisions. Second, it sought to identify combinations of attributes and levels associated with lower perceived constraints on participation. Third, it examined participant segmentation based on the relative importance assigned to each constraint attribute.

Conjoint analysis estimates the contribution of multiple attributes when individuals evaluate alternatives presented simultaneously, making it suitable for investigating how individuals integrate multiple constraints in participation decision-making. The validity of this approach depends on the careful specification of attributes and their corresponding levels (Agarwal et al., 2015). In conjoint research, attributes represent key evaluative characteristics considered in decision-making, whereas levels denote the specific values that operationalize each attribute within the research design (Jang & Baek, 2024). These elements should reflect actual decision criteria, be theoretically grounded, and remain perceptible and measurable to respondents (Rao, 2014).

In this study, attributes were defined as perceived constraints on exercise participation, grounded in the intrapersonal, interpersonal, and structural dimensions of constraint theory. Although conceptually distinct, these constraints are typically evaluated concurrently during participation decisions. Accordingly, the analytical design decomposed each constraint into independent attributes to estimate its relative importance and to derive attribute combinations associated with lower perceived constraints. To identify appropriate attributes and levels, a multi-stage procedure was implemented.

In the first stage, an open-ended survey was conducted to explore perceived constraints experienced in recent participation contexts. Second, prior research on exercise participation constraints was systematically reviewed to refine theoretical categories. Third, the preliminary items were refined through expert consultation with researchers in sport psychology and exercise behavior. The experts reviewed the preliminary attributes and levels for conceptual clarity, theoretical relevance, overlap among attributes, and suitability for conjoint analysis. Based on their feedback, redundant or ambiguously defined items were removed, and only those clearly relevant to participation decision-making were retained as final attributes and levels (Blamey et al., 2002). This structured procedure enhances methodological rigor and supports subsequent segmentation based on relative importance estimates.

In the second stage, a systematic literature review was conducted to identify constraints to exercise participation, with relevant attributes and levels derived primarily from academic research.

Previous studies have identified a wide range of constraints that hinder participation in exercise and recreational sport activities. Lin et al. (2022), in a study of urban residents in China, classified constraints into multiple dimensions: intrapersonal factors such as physical problems, psychological barriers, negative attitudes toward fitness, and lack of interest; interpersonal factors such as lack of partners and negative attitudes from family and friends; environmental constraints, including adverse weather, safety concerns, and poor hygiene; leisure opportunity constraints such as lack of time, limited activity options, and high participation costs; and facility and service management constraints, including inadequate programs, lack of professional management, and insufficient information. Similarly, Ntovoli et al. (2025) categorized exercise constraints into structural constraints (time, facilities, and cost), intrapersonal constraints (psychological factors, lack of interest, and prior experience), and interpersonal constraints (lack of partners and lack of knowledge).

Expanding on this perspective, Crossman et al. (2024) proposed a broader multilevel framework encompassing individual factors (health conditions, personal circumstances, and self-beliefs), interpersonal influences (family, peers, and social norms), organizational factors (sport structures and resources), community-level constraints (access and cultural norms), and policy-related factors (program delivery). In a more parsimonious approach, Koronios and Kriemadis (2018) highlighted key constraints, including facilities and accessibility, knowledge, time, interest, social partners, and individual characteristics. Earlier studies have also emphasized specific barriers. Spivey and Hritz (2013) identified self-consciousness during exercise, lack of equipment, insufficient knowledge, time constraints, competing demands from significant others, and lack of social support. Similarly, Y. K. Kim and Trail (2010) distinguished between internal constraints—including lack of knowledge, absence of companions, and low interest—and external constraints such as time commitments, cost, alternative leisure options, and accessibility issues.

The theoretical foundations for these findings can be traced to Crawford and Godbey (1987), who conceptualized leisure constraints into three primary categories: intrapersonal, interpersonal, and structural. This framework has been widely adopted and extended in subsequent research. Consistent with this tradition, M. K. Kim et al. (2015) identified health, social factors, cost, and time as key determinants of participation constraints, while D. Cho and Price (2016) reaffirmed the tripartite structure of intrapersonal, interpersonal, and structural constraints. Furthermore, Park et al. (2020) highlighted lack of interest, lack of confidence, insufficient information, limited facilities and accessibility, and time constraints as critical barriers to exercise participation.

The exercise participation constraint attributes identified through these two processes were finalized following expert consultation. Consequently, six key constraint attributes influencing exercise participation were identified: intrapersonal constraints, comprising interest in exercise and physical fatigue; interpersonal constraints, comprising partner availability and social-norm pressure; and structural constraints, comprising cost burden and time burden. The final six attributes were selected because they represented the most frequently identified and theoretically relevant constraints across the open-ended responses, literature review, and expert consultation. The number of attributes was also limited to six to maintain respondent manageability in the conjoint task. Each attribute was operationalized using two levels to ensure conceptual clarity, reduce task complexity, and allow respondents to compare profiles based on clearly distinguishable constraint conditions (see Table 3 and Table 4).

In conducting conjoint analysis, attributes and their corresponding levels must be conceptually distinct and mutually exclusive to prevent overlap (Hair et al., 1998). Such differentiation is critical for accurately capturing realistic decision-making contexts (Jang & Baek, 2024). In accordance with these principles, the present study systematically operationalized attribute levels related to exercise participation constraints; the final configuration is presented in Table 2.

**Table 2:** Open-ended survey questions on exercise participation constraints.

Question	Response Type	Findings
During the past three months, have you ever planned to exercise but not done so?	Yes/No	71% Yes/29% No
The main reasons that discourage you from exercising	Open-ended	Lack of time to exercise Muscle soreness/minor injuries Feeling too tired after work Financial cost of exercising Difficulty maintaining interest in exercise Absence of exercise partners Environmental concerns (e.g., weather conditions, facility accessibility) Lack of exercise knowledge Irregular personal schedules Work or study pressure/overtime work Declining exercise motivation Boredom from repetitive exercise routines

**Table 3:** Frequent response of open-ended survey and literature review findings.

Open-Ended Survey	Literature Review
Time constraint (e.g., personal schedules, work, child care) Physical fatigue and injury concerns Cost burden related to exercise participation Difficulty maintaining interest in exercise Absence of exercise partners Environmental constraints (e.g., weather conditions, facility accessibility)	Time constraint (Lack of time) Cost/Financial constraint Lack of Interest/motivation Fatigue/Physical (Health) condition Lack of partners Lack of exercise knowledge

**Table 4:** Exercise participation constraints’ attributes and the level.

Constraints Dimension	Constraints’ Attributes	Constraints’ Level
Intrapersonal constraints	Interest in exercise	High interest
		Low interest
	Physical fatigue	Rarely tired
		Easily tired
Interpersonal constraints	Partner availability	Available
		Not available
	Social norm pressure	Low pressure
		High pressure
Structural constraints	Cost burden	Low-cost burden
		High-cost burden
	Time burden	Sufficient time to exercise
		Insufficient time to exercise

Following the selection of attributes and levels related to exercise participation constraints, stimulus profiles were constructed for respondent evaluation. Common approaches to profile development include paired comparisons, trade-off techniques, and the full-profile method. The present study adopted the full-profile approach, as it presents all selected attributes simultaneously and reflects the complexity of participation decision-making contexts (Blamey et al., 2002) (see Table 5).

Because including multiple attributes can yield many possible combinations, fractional factorial designs are typically used to reduce respondent burden while preserving the ability to estimate main effects. In this study, six constraint attributes, each with two levels, yielded 64 possible combinations (2 × 2 × 2 × 2 × 2 × 2). To ensure manageable cognitive demands and statistical efficiency, an orthogonal fractional factorial design was employed, resulting in a reduced set of 16 profiles for evaluation (Dean, 2004). Orthogonal arrays were generated using SPSS Window Ver. 25.0 software to maintain statistical balance and independence among attributes.

Participants were instructed to rank the 16 profiles according to the perceived degree of participation constraints, ranging from the least restrictive profile combination to the most restrictive. The ranking data were analyzed to estimate the relative importance of each constraint attribute and the part-worth utility values associated with each level. In this study, these values were interpreted as indicating how respondents prioritized the perceived constraints within the survey task. They were not interpreted as direct measures of stable cognitive mechanisms or actual exercise behavior.

### 2.3. Instrument

This study examines how the cognitive weighting of exercise participation constraints varies across levels of exercise involvement. To enable this group-based analysis, exercise involvement was measured using a six-item scale adapted from Kang and Hong (2025). Participants evaluated each item on a five-point Likert scale ranging from 1 (strongly disagree) to 5 (strongly agree). As exercise involvement served as the basis for group classification and was the only construct measured using multiple items in this study, its validity and reliability were assessed. Although the scale was expected to be unidimensional, exploratory factor analysis (EFA) was conducted as a preliminary check to confirm its dimensionality before using the mean involvement score for group classification.

An exploratory factor analysis (EFA) using principal component analysis with Varimax rotation was conducted to examine the construct validity of the scale. The results supported a unidimensional structure, with all six items loading on a single factor. The extracted factor accounted for 68.06% of the total variance, and factor loadings ranged from 0.743 to 0.865, indicating strong relationships between the observed items and the underlying construct. The Kaiser–Meyer–Olkin (KMO) measure of sampling adequacy was 0.864, and Bartlett’s test of sphericity was statistically significant (χ^2^ = 742.318, *df* = 15, *p* < 0.001), confirming the suitability of the data for factor analysis. Reliability analysis indicated satisfactory internal consistency, with a Cronbach’s α of 0.859. These results demonstrate that the exercise involvement scale possesses acceptable construct validity and reliability for use in this study(see Table 6).

Respondents were classified into high and low exercise involvement groups based on the overall mean score of exercise involvement (3.84), which was used as the cut-off value (see Table 7). The mean split was used for exploratory group-based comparison because the purpose of this study was to examine whether constraint-weighting patterns differed between relatively higher and lower exercise involvement groups. This approach allowed the same conjoint procedure to be applied separately to the two involvement groups and enabled a straightforward comparison of relative importance structures. However, the mean score was not assumed to represent a definitive psychological boundary between qualitatively distinct involvement types. Rather, it was used as a practical classification criterion for exploratory comparison within the present sample. Those scoring at or above the mean were categorized as the high exercise involvement group, whereas those scoring below the mean were categorized as the low exercise involvement group. Among the total 283 respondents, 62.2% (n = 176) were classified into the high involvement group (M = 4.29), and 37.8% (n = 107) into the low involvement group (M = 3.22).

### 2.4. Data Analysis

The data were analyzed in several steps. First, conjoint analysis was performed using the total sample to estimate the part-worth utilities and relative importance values of the perceived exercise participation constraint attributes. The conjoint analysis was conducted using the ranking-based conjoint procedure in SPSS Window Ver. 25.0 and focused on main effects. Interaction effects were not estimated, and holdout profiles were not included because the study aimed to examine relative importance structures rather than predict choices for validation profiles. Although ranking fatigue was not formally assessed, the profile set was reduced from 64 possible combinations to 16 profiles to reduce respondent burden. Pearson’s R was interpreted only as an indicator of internal correspondence between observed and estimated rankings within the conjoint task, not as evidence of predictive validity or behavioral realism. Second, the same conjoint procedure was applied separately to the exercise involvement groups described above to compare differences in constraint weighting according to involvement level. Third, K-means cluster analysis was conducted using the relative importance values derived from the conjoint analysis to identify participant segments with similar constraint-weighting patterns. One-way ANOVA was then used to examine whether constraint importance differed across clusters, and chi-square tests were conducted to compare demographic and exercise-related characteristics among the identified clusters.

## 3. Results

### 3.1. The Relative Importance of Exercise Participation Constraints

The aggregate conjoint analysis revealed that “interest in exercise” was the most influential constraint affecting participation, accounting for 26.412% of the total relative importance. Within this attribute, a high interest level yielded a positive part-worth utility value (0.842), indicating that strong interest substantially mitigates perceived constraints. Conversely, a low interest level showed a negative utility value (−0.842), representing a significantly stronger barrier to exercise. Physical fatigue emerged as the second most influential attribute (20.103%). “Rarely feeling tired” was associated with reduced participation constraints (0.615), whereas “easily feeling tired” resulted in a negative utility value (−0.615), indicating a greater perceived restriction. The third most significant attribute was time burden (17.284%). Having sufficient time was linked to lower perceived constraints (0.502), while insufficient time (−0.502) was associated with greater restrictions. Cost burden followed in importance at 13.187%. Low cost (0.341) reduced perceived constraints, while high cost (−0.341) increased them. Partner availability ranked fifth (12.004%), where the presence of a partner (0.294) lowered constraints, and the absence of one (0.294) heightened them. Finally, social norm pressure showed the lowest relative importance (11.010%). Low social pressure (0.158) was perceived as less restrictive, while high pressure (−0.158) was linked to stronger constraints. Substantively, the difference between the highest-ranked attribute, interest in exercise, and the lowest-ranked attribute, social norm pressure, suggests that intrapersonal constraints carried considerably greater weight than interpersonal constraints within the conjoint task. The relatively high importance of physical fatigue also indicates that perceived bodily burden was a meaningful factor in how recent adult exercisers evaluated participation constraints.

Pearson’s correlation coefficient indicated acceptable internal correspondence between the observed and estimated rankings (r = 0.871, *p* < 0.001). The results of the relative importance are shown in Table 8.

### 3.2. Constraint Importance by Exercise Involvement

The subsequent analysis examined the relative importance of constraints on exercise participation across groups categorized by their level of exercise involvement.

For the high-involvement group, conjoint analysis identified “time burden” as the most critical constraint, accounting for 28.192% of the total relative importance. Within this attribute, having sufficient time (0.873) was strongly associated with diminished perceived constraints, whereas a lack of time (−0.873) acted as a substantial barrier to participation. The second most influential attribute was “interest in exercise,” representing 19.236% of the overall importance. Participants with high exercise interest (0.611) perceived significantly fewer barriers, while low interest (−0.611) was linked to heightened restrictions.

“Cost burden” ranked third, accounting for 17.842% of the total importance. Low costs (0.416) were found to mitigate participation constraints, whereas high costs (−0.416) exacerbated the perceived difficulty of engaging in exercise. Physical fatigue followed in fourth place, with a relative importance of 14.358%. Respondents who rarely felt fatigued (0.284) reported lower perceived constraints, while those prone to exhaustion (−0.284) felt more restricted. The fifth attribute, “partner availability,” accounted for 11.127% of the total importance. The presence of an exercise partner (0.192) served to lower perceived barriers, whereas the absence of a partner (−0.192) increased them. Finally, “social norm pressure” exhibited the lowest relative importance (9.245%), where low social pressure (0.134) was viewed as less restrictive compared to high pressure (−0.134).

Pearson’s correlation coefficient indicated acceptable internal correspondence between the observed and estimated rankings for the high-involvement group (r = 0.855, *p* < 0.001). The results of the relative importance are shown in Table 9.

Conjoint analysis for the low exercise involvement group (n = 107) revealed that “interest in exercise” was the most predominant constraint, accounting for 29.384% of the total relative importance. Within this attribute, a high interest level exhibited a positive part-worth utility value (0.912), suggesting that strong interest significantly mitigates perceived participation barriers. Conversely, low interest (−0.912) was associated with a markedly higher level of perceived restriction. The second most influential attribute was “cost burden” (18.917). Low costs (0.563) were linked to reduced participation constraints, whereas high costs (−0.563) intensified the perceived difficulty of engaging in exercise. “Time burden” ranked third, representing 16.900% of the total importance. Having sufficient time (0.472) was correlated with lower perceived constraints, while insufficient time (−0.472) acted as a stronger deterrent. The fourth attribute, “physical fatigue,” held a relative importance of 14.173%. Respondents who rarely felt tired (0.421) reported lower perceived barriers, whereas those susceptible to fatigue (−0.421) faced greater restrictions. “Partner availability” followed in fifth place (11.624), where the presence of an exercise partner (0.248) helped lower constraints, and the absence of one (−0.248) heightened them. Finally, “social norm pressure” was the least influential attribute (9.002); low social pressure (0.152) was viewed as relatively less restrictive, while high pressure (−0.152) corresponded to stronger perceived constraints.

Pearson’s correlation coefficient indicated acceptable internal correspondence between the observed and estimated rankings for the low-involvement group (r = 0.846, *p* < 0.001). The results of the relative importance are shown in Table 10.

Taken together, these results suggest that high-involvement exercisers prioritized structural feasibility, particularly time burden, whereas low-involvement exercisers prioritized motivational or affective barriers, particularly lack of interest.

### 3.3. Participant Segmentation Based on Relative Importance of Constraints

In this study, participant segmentation was conducted by applying K-means cluster analysis to the relative importance values of exercise participation constraints derived from the conjoint analysis. The four-cluster solution was selected because it provided interpretable and distinct patterns in the relative importance values of the constraint attributes. However, because objective validation procedures such as silhouette statistics, the elbow method, information criteria, or replication with an independent sample were not applied, the cluster solution should be interpreted as an exploratory grouping within the present sample. To examine whether the importance of constraints differed significantly across clusters, a one-way analysis of variance (ANOVA) was subsequently performed.

The results indicated statistically significant differences among the clusters for interest in exercise (F = 56.381), physical fatigue (F = 47.952), time burden (F = 53.164), and cost burden (F = 44.289) (*p* < 0.001). Partner availability also showed a statistically significant difference across clusters (F = 3.218, *p* < 0.05), although its influence was relatively small compared with the other constraint attributes. Social norm pressure did not show a statistically significant difference across clusters, suggesting that its role in distinguishing the clusters was relatively limited compared with other constraint attributes.

An examination of cluster characteristics revealed distinct constraint profiles among participants. Cluster 1 (n = 72) was characterized by the highest importance assigned to interest in exercise (34.218). Cluster 2 (n = 65) showed the greatest importance for physical fatigue (29.784). Cluster 3 (n = 77) assigned the highest importance to time burden (31.642). Finally, Cluster 4 (n = 69) emphasized cost burden (28.507) as the most prominent constraint attribute.

Although six attributes were included in the analysis, the clustering results converged into four groups based on similarities in the relative importance values of the constraint attributes rather than the number of attributes themselves. The results of cluster analysis are shown in Table 11.

To further segment participants and examine the characteristics of each cluster identified through the cluster analysis, demographic variables, including gender, age, weekly exercise frequency, and exercise participation duration, were incorporated into a chi-square test.

Results showed that gender did not show a statistically significant difference across clusters (χ^2^ = 2.960, *p* = 0.398). In contrast, age (χ^2^ = 19.600, *p* = 0.021), weekly exercise frequency (χ^2^ = 30.291, *p* < 0.001), and exercise participation duration (χ^2^ = 24.686, *p* = 0.003) showed statistically significant differences among the clusters.

A closer examination of these distributions revealed that Cluster 1 consisted of a relatively higher proportion of participants in their twenties and those with shorter exercise participation histories. Cluster 2 was characterized by a larger share of participants with moderate weekly exercise frequency, whereas Cluster 3 was distinguished by individuals who exercise frequently, including daily participants. Notably, Cluster 4 included a comparatively larger proportion of older participants and individuals with extensive exercise experience. The results of the chi-square test are shown in Table 12.

Based on the results of the chi-square test examining differences among clusters using demographic variables, participants were classified into four distinct groups characterized by specific demographic profiles.

Cluster 1 showed a relatively higher proportion of participants in their 20s. In terms of weekly exercise frequency, the largest proportion of participants exercised 1–2 times per week. Regarding exercise participation duration, participants with 6 months to less than 1 year of experience accounted for a relatively higher proportion in this cluster. Cluster 2 was characterized by a relatively higher proportion of participants in their 40s. In terms of weekly exercise frequency, participants exercising 3–4 times per week accounted for the largest proportion. Regarding exercise participation duration, participants with 1 to less than 3 years of exercise experience represented a relatively higher share in this cluster. Cluster 3 showed a higher proportion of participants in their 30s. In terms of weekly exercise frequency, participants who exercised every day represented a relatively larger proportion. In addition, participants with 3 years or more of exercise participation experience accounted for a comparatively higher proportion in this cluster. Cluster 4 included a relatively higher proportion of participants aged 50 years or older. Regarding weekly exercise frequency, participants exercising 3–4 times per week represented the largest proportion. In terms of exercise participation duration, participants with 3 years or more of exercise experience accounted for a relatively higher proportion in this cluster. The cluster labels were assigned as descriptive summaries based on the dominant constraint attribute and the observed demographic and exercise-related characteristics of each cluster. These labels should not be interpreted as indicating developmental stages, causal pathways, or empirically verified behavioral typologies(see Table 13).

## 4. Discussion

### 4.1. Discussion of Constraint Importance Analysis

This study discusses the findings derived from the relative importance analysis of perceived exercise participation constraint attributes among adults with recent exercise participation experience. In interpreting these findings, the estimated importance values should be understood as inferred evaluative weights derived from the conjoint ranking task, rather than as direct evidence of stable cognitive mechanisms.

In the aggregate sample, the priority of importance was identified in the following order: exercise interest, physical fatigue, time constraints, financial burden, presence of a partner, and social normative pressure. For the exercise high-involvement group, the order was time constraints, exercise interest, financial burden, physical fatigue, presence of a partner, and social normative pressure. Conversely, the exercise low-involvement group prioritized exercise interest, followed by financial burden, time constraints, physical fatigue, presence of a partner, and social normative pressure.

Interpreting these results through the lens of the constraint framework, the aggregate sample exhibited a hierarchical sequence of intrapersonal constraints (exercise interest, physical fatigue) > structural constraints (time constraints, financial burden) > interpersonal constraints (presence of a partner, social normative pressure). Within this sample, this pattern suggests that psychological and emotional factors may play a relatively greater role than physical and contextual barriers in forming perceived constraints on exercise participation decisions. This aligns with the theoretical assertions of Rhodes et al. (2017), who argued that psychological motivational states and affective responses serve as core determinants in shaping physical activity behavior. Furthermore, the primacy of intrapersonal constraints at the aggregate level implies that perceived participation constraints may be more strongly associated with internal psychological states than with external structural conditions. This result is theoretically congruent with Self-Determination Theory (SDT), which posits that autonomous motivation originating from within the individual serves as a more robust predictor of sustained behavioral engagement than motivation driven by external controls (Ryan & Deci, 2000). However, because this study did not directly measure motivational regulation styles, the SDT-based interpretation should be understood as a theoretical reference point rather than as direct empirical evidence of specific motivational processes. In addition, because the present study was conducted among adults who had already participated in exercise during the previous three months, this result should be interpreted as reflecting perceived constraint weighting among recent exercisers rather than as evidence of exercise barriers in the general population or among non-participants.

Notably, unlike the aggregate trend, time constraints emerged as the most significant factor for the exercise high-involvement group, with exercise interest ranking second. Within the constraint typology, this group demonstrated an intersection of intrapersonal and structural constraints in the following order: time constraints (first), exercise interest (second), financial burden (third), and physical fatigue (fourth). Specifically, with structural constraints (time and cost) flanking the intrapersonal constraints (interest and fatigue), the relative weight of structural barriers increased significantly compared to the total group. Individuals classified into the exercise high-involvement group are characterized by high perceptions of the value, necessity, and enjoyment of exercise. This profile suggests that their internalized motivation for physical activity is already well-established. Consequently, for individuals whose intrinsic motivation is internalized, the salient barriers are no longer rooted in motivation formation but rather in the structural and physical allocation of time and financial resources required for habitual participation. This interpretation resonates with the findings of J. F. Sallis and Saelens (2000) and Trost et al. (2002), which identified lack of time as one of the most consistently reported structural barriers across diverse populations. These results indicate that once motivational prerequisites are met, the relative weight of time constraints becomes particularly pronounced, reflecting a shift in the primary locus of constraints from the psychological domain to the behavioral execution stage. Additionally, the third-place ranking of financial burden in this group suggests that economic accessibility functions as a secondary structural barrier among motivated participants, consistent with existing literature (R. E. Sallis et al., 2016). Nevertheless, this interpretation should be limited to adult exercisers with recent participation experience, as the present sample did not include non-participants or individuals who had completely discontinued exercise.

In contrast, the exercise low-involvement group maintained a structure where intrapersonal constraints were dominant, with exercise interest being the most influential barrier, followed by financial and time constraints. Interpreting this through the typology framework, the sequence was exercise interest (first), financial burden (second), time constraints (third), and physical fatigue (fourth). While structural constraints were concentrated in the middle rankings, the placement of intrapersonal constraints (interest and fatigue) at the top and bottom of the hierarchy confirms that, similar to the aggregate sample, internal factors remain the core determinants of participation decisions. This pattern suggests that the primary barrier for individuals in the exercise low-involvement group is not the absence of structural opportunities, but rather an insufficient formation of motivational orientation. Their barriers are more accurately characterized as motivational deficits rather than physical obstacles. This conceptualization is theoretically grounded in the motivation continuum described by Ryan and Deci (2000).

These findings are also consistent with empirical evidence from Teixeira et al. (2012), which identified autonomous motivation—specifically intrinsic motivation—as a significant predictor of long-term physical activity adherence. Therefore, efforts to promote exercise among the low-exercise involvement group may need to focus on cultivating intrinsic motivation rather than merely reducing structural barriers. Structural interventions alone may be insufficient to trigger behavioral change until a certain threshold of internal motivation is achieved. For these individuals, psycho-behavioral strategies aimed at increasing the perceived benefits and lowering the perceived psychological barriers of exercise may be required. However, because this study did not directly measure motivation, self-efficacy, or goal orientation, these practical implications should be interpreted cautiously and regarded as tentative suggestions for future intervention design.

Meanwhile, interpersonal (social) constraints, such as the presence of a partner and social normative pressure, consistently ranked lowest (fifth and sixth) across all groups. Within the present sample, this indicates that exercise participation is governed more strongly by internal factors and structural conditions than by relational contexts or social expectations. While Crawford et al.’s (1991) hierarchical model of leisure constraints suggests that interpersonal constraints precede structural ones, our results show their influence to be relatively limited in the exercise context. This may reflect the modern trend of exercise being perceived as an individualized behavior (Downward & Rasciute, 2010).

Following the identification of these constraints, it may be useful to consider constraint negotiation strategies to mitigate barriers and enhance participation. Constraint negotiation refers to the proactive cognitive and behavioral efforts individuals employ to modify or overcome perceived barriers rather than succumb to them (Hubbard & Mannell, 2001). For the aggregate and exercise low-involvement groups, cognitive negotiation strategies may be particularly relevant. This involves reappraising perceived constraints through attitudinal restructuring and re-evaluating the value and relevance of exercise. Since establishing interest and positive affect is a prerequisite for the exercise low-involvement group, strategies that support interest, perceived value, and positive attitudes toward exercise may be useful. Strategies such as self-monitoring and incremental mastery experiences may also help mitigate intrapersonal constraints by enhancing perceived competence and self-efficacy (Cugelman, 2013). However, these implications should be interpreted cautiously because the present study did not directly test specific behavioral interventions.

Conversely, for the exercise high-involvement group, behavioral negotiation strategies may be particularly useful to address structural constraints, particularly time. This involves actively restructuring behavioral routines and resource utilization. Strategies such as rescheduling, “time-splitting” (segmenting exercise bouts), and integrating physical activity into existing daily routines have been reported as effective for maintaining adherence (J. F. Sallis & Saelens, 2000). Furthermore, utilizing low-cost exercise alternatives and community-based resources may be considered as complementary options to alleviate financial burdens. These implications are most applicable to adults with recent exercise experience and should not be generalized to physically inactive populations without further empirical verification.

### 4.2. Discussion of Participant Segmentation

The following section discusses the results of the participant segmentation. The analysis delineated four distinct clusters, each characterized by unique demographic profiles—including age, weekly exercise frequency, and duration of exercise participation—as well as primary constraint factors. These clusters were conceptualized as: ‘Interest-constrained beginners’, ‘Fatigue-sensitive participants’, ‘Time-constrained active participants’, and ‘Cost-sensitive experienced participants’. The segmentation results suggest that adults with recent exercise participation experience were not homogeneous in their perceived constraint structures. Consistent with the exploratory nature of the cluster analysis described above, these segments should be interpreted as descriptive groupings rather than stable behavioral typologies.

The first cluster, ‘Interest-constrained beginners’, primarily consists of participants in their 20s with a short duration of exercise participation and low frequency, representing a typical exercise low-involvement group. Their most significant barrier, a ‘lack of interest in exercise,’ may be interpreted as reflecting relatively weak intrinsic interest from the perspective of Self-Determination Theory (Ryan & Deci, 2000), although motivational regulation styles were not directly measured in this study. This finding is consistent with previous studies showing that lack of interest, low motivation, and negative attitudes toward fitness are major intrapersonal constraints to exercise and recreational sport participation (Lin et al., 2022; Ntovoli et al., 2025; Park et al., 2020). Lin et al. (2022) identified lack of interest and negative attitudes toward fitness as important intrapersonal barriers, while Ntovoli et al. (2025) classified lack of interest and psychological factors as key intrapersonal constraints. The present finding extends previous research by showing that lack of interest is especially salient among relatively younger and less experienced adult exercisers. Since beginners in their 20s often perceive exercise as a strenuous or obligatory task, a psych-behavioral approach is required to counteract these perceptions. Rather than merely emphasizing the health benefits of exercise, it may be helpful to frame exercise as an enjoyable and personally relevant leisure activity. To achieve this, broad strategies that reduce the psychological burden of the initiation stage and facilitate habit formation may be considered. Furthermore, setting simple and attainable goals rather than overly ambitious plans may help alleviate the pressure of exercise and allow for the accumulation of successful experiences. Simultaneously, creating supportive exercise environments that reduce reliance on willpower alone may help lower the psychological burden of exercise initiation. Such an approach may help individuals perceive exercise as a more manageable part of daily life rather than as a task requiring significant resolve. However, because the present study did not directly test specific environmental or behavioral intervention strategies, this implication should be interpreted as a general direction for addressing intrapersonal constraints among the exercise low-involvement group.

The second cluster is the ‘Fatigue-sensitive participants’, primarily composed of individuals in their 40s. Despite being moderate participants who maintain a frequency of 3–4 times per week, they identify ‘physical fatigue’ as their primary constraint. This finding is broadly consistent with previous research emphasizing physical problems, health conditions, and fatigue-related factors as important constraints to physical activity participation (Crossman et al., 2024; Lin et al., 2022; Park et al., 2020). Crossman et al. (2024) suggested that individual-level factors, including health conditions and personal circumstances, can shape sport participation, while Park et al. (2020) highlighted health-related barriers among adults with physical conditions. The present study adds to this line of research by suggesting that fatigue may remain a central perceived constraint even among adults who already participate in exercise regularly. Their fatigue is likely a complex constraint coupled with issues of energy allocation in daily life rather than a simple lack of physical fitness. Therefore, this group may benefit from flexible and recovery-oriented exercise options that reduce the perceived burden of participation. Specifically, lower-intensity, shorter-duration, or adaptable exercise options may be considered so that exercise is not perceived as an additional burden. Additionally, on days with poor physical condition, flexible intensity adjustments or light-activity alternatives may help reduce psychological and physical burdens. By integrating exercise into daily life in a manageable way, exercise may be perceived as a ‘means of vitality recharge’ rather than a ‘task’ requiring excessive energy expenditure. However, this interpretation should be approached with caution because fatigue was measured as a perceived constraint in the conjoint task, not as a clinically assessed physical condition.

The third cluster, ‘Time-constrained active participants’, consists of individuals in their 30s who exercise daily and are classified as a quintessential exercise high-involvement group. In this group, where intrinsic motivation is well-established, the locus of constraints has shifted from the psychological domain to the behavioral execution stage, specifically, ‘time constraints.’ This result is consistent with previous studies identifying lack of time as one of the most frequently reported structural constraints to exercise and sport participation (Koronios & Kriemadis, 2018; Lin et al., 2022; J. F. Sallis & Saelens, 2000; Trost et al., 2002). Koronios and Kriemadis (2018) identified time as a key constraint in sport and exercise events, while J. F. Sallis and Saelens (2000) and Trost et al. (2002) also emphasized time-related barriers in physical activity research. The present finding suggests that even highly active participants may continue to experience time burden as a major barrier to maintaining exercise participation. They face a strategic challenge: not “why should I exercise,” but “how can I sustain it within a busy daily life.” Thus, active behavioral negotiation strategies, such as time-splitting or integrating physical activity into daily routines, may be useful. For instance, flexible time-management strategies, shorter exercise sessions, or the use of accessible exercise environments may help reduce perceived time burden. These methods may provide general directions for high-involvement participants in their 30s to maintain their habits without losing momentum. However, these suggestions should be interpreted as general implications derived from the observed importance of time constraints, rather than as intervention strategies directly tested in this study.

The fourth cluster, ‘Cost-sensitive experienced participants’, is composed of individuals aged 50 and older who have maintained stable exercise habits for over three years but identify ‘cost burden’ as their primary constraint. This finding is consistent with previous studies that identified financial cost and price sensitivity as important structural constraints to leisure sport and exercise participation (S.-Y. Kim, 2024; Koronios & Kriemadis, 2018; Rao, 2014). S.-Y. Kim (2024), in particular, showed that price sensitivity can be an important factor in leisure sport participation among older adults. Similarly, Lin et al. (2022) and Y. K. Kim and Trail (2010) identified cost as an external or structural barrier to participation. The present study extends these findings by showing that cost burden may be especially salient among experienced older adult exercisers, even when they have already established stable exercise habits. This indicates that for age groups approaching retirement or preparing for an active later life, economic sustainability serves as a critical determinant for maintaining physical activity. Despite having a long duration of exercise participation, there is a risk of dropout when facing structural barriers like economic accessibility. Therefore, strategies that improve economic accessibility, such as low-cost or community-based exercise opportunities, may be useful for supporting continued exercise participation in this group. However, because this study did not directly test cost-reduction interventions, this implication should be interpreted cautiously. However, because this study did not collect detailed information on income, employment status, physical and mental health status, diagnosed diseases or pathologies, or living area, this interpretation should be considered exploratory.

In summary, the discussion of participant segmentation highlights the importance of differentiated constraint negotiation strategies tailored to the characteristics of each group. Such a multifaceted approach may provide a behavior-centered guideline for understanding and addressing perceived exercise participation constraints among adult exercisers. Overall, the segmentation results are consistent with previous research suggesting that exercise and sport participation constraints are multidimensional and vary across individuals according to psychological, structural, and contextual conditions (Crossman et al., 2024; Koronios & Kriemadis, 2018; Lin et al., 2022; Ntovoli et al., 2025). The present study contributes to this literature by showing that adult exercisers can be segmented according to the relative importance they assign to different perceived constraints, rather than by the mere presence or absence of individual barriers. Accordingly, the practical implications of the segmentation analysis should be interpreted as broad directions suggested by perceived constraint patterns, rather than as specific intervention prescriptions. Because this study did not directly test behavioral intervention strategies, future research should examine whether strategies tailored to dominant perceived constraints actually improve exercise participation or adherence. However, given the limited sample characterization, the absence of information on physical and mental health status, diagnosed diseases or pathologies, and living area, and the exclusion of non-participants, the implications of this study should be interpreted within the empirical scope of the present sample.

## 5. Conclusions

### 5.1. Summary of Findings and Implications

This study examined how adults with recent exercise participation experience the cognitively weighing of different perceived constraints on exercise participation and whether these weighting structures can serve as a basis for meaningful segmentation. The sample comprised adults aged 19 and older who had engaged in exercise at least once per week for a minimum of 30 min during the preceding three months. Three objectives guided the analysis: to identify the relative importance of perceived exercise participation constraints among adult exercisers, to examine whether constraint prioritization differs according to exercise involvement level, and to segment participants based on the importance assigned to individual constraint attributes.

The findings indicate that, within this sample, perceived exercise participation constraints are not weighted uniformly across individuals. At the aggregate level, intrapersonal factors—particularly interest in exercise and physical fatigue—emerged as the most influential perceived constraints, followed by structural factors such as time burden and cost burden. In contrast, interpersonal and social dimensions, including partner availability and social norm pressure, were assigned relatively lower importance.

Constraint prioritization was also found to vary according to exercise involvement level. Among highly involved adult exercisers, time burden emerged as the most critical perceived constraint, whereas for those with lower involvement, interest in exercise was the most salient factor. This contrast suggests that constraint perception is not fixed but varies according to individuals’ level of engagement and participation context among adults who already have recent exercise participation experience.

The cluster analysis identified four distinct participant segments based on constraint importance structures: interest-constrained beginners, fatigue-sensitive participants, time-constrained active participants, and cost-sensitive experienced participants. This finding indicates that adult exercisers with recent participation experience are heterogeneous and that their *perceived* constraint profiles cannot be adequately explained by a single, unified pattern.

The chi-square analysis further supported this segmentation by revealing significant differences across clusters in demographic and behavioral characteristics, including age, weekly exercise frequency, and participation experience. Participants with lower exercise frequency and shorter participation histories were more likely to belong to segments characterized by motivational constraints, whereas those with higher frequency and longer experience were more likely to be associated with structural constraints such as time and cost. However, these patterns should be interpreted within the scope of the present sample and should not be generalized to the general population or to non-participants.

Taken together, these findings suggest that among adults with recent exercise participation experience, perceived exercise participation constraints can be understood as a multidimensional construct whose relative importance varies across individuals and groups within a conjoint evaluation task. This study contributes to the literature by applying conjoint analysis to capture the relative importance of multiple perceived constraints simultaneously and by providing a segmentation-based perspective on constraint perception among adult exercisers.

From a practical perspective, the findings suggest that strategies to promote continued exercise participation among adult exercisers may benefit from differentiated approaches tailored to the dominant perceived constraints of each segment. For example, interventions targeting low-involvement adult exercisers may focus on enhancing interest and motivation, whereas strategies for highly involved adult exercisers may prioritize reducing time-related constraints. In addition, segment-specific approaches addressing fatigue and cost-related factors may further improve the effectiveness of participation maintenance efforts. These implications should be interpreted cautiously because the present study did not include non-participants and did not account for all health-related or contextual factors that may shape perceived exercise constraints. Therefore, the findings should be understood as exploratory evidence of perceived constraint prioritization among recent adult exercisers rather than as definitive behavioral typologies or confirmed intervention guidelines.

### 5.2. Limitations

This study has several limitations that should be acknowledged.

First, the sample was limited to adults aged 19 years or older who had participated in exercise at least once per week for at least 30 min during the previous three months. Therefore, the findings should be interpreted as reflecting perceived exercise participation constraints among adult exercisers with recent exercise participation experience rather than constraints experienced by the general adult population or by non-participants. Because individuals who were physically inactive or who had discontinued exercise were not included, the results cannot be generalized to non-participants or to the broader population of adults with varying levels of physical activity. This limitation is particularly important because individuals who do not currently exercise may experience more severe participation constraints than the respondents included in this study. In addition, because data were collected through an online survey platform using non-probability quota sampling, the sample may have overrepresented individuals who were digitally engaged, accessible through online panels, or relatively health-conscious. As a result, the findings should be interpreted as exploratory evidence of perceived constraint prioritization among recent adult exercisers rather than as representative evidence of exercise participation barriers in the broader adult population.

Second, the characterization of the sample was limited to demographic and exercise-related variables, including gender, age, weekly exercise frequency, and duration of exercise participation. Although these variables provided useful information for segmenting adult exercisers, the study did not collect detailed information on physical health status, mental health status, diagnosed diseases or pathologies, living area, facility accessibility, income, employment status, or other contextual conditions that may influence perceived exercise participation constraints. These unmeasured factors may have affected how respondents evaluated fatigue, time, cost, and other constraint attributes. Future research should include more comprehensive health-related, socioeconomic, and environmental variables to better explain differences in constraint perception.

Third, this study used a ranking-based conjoint design with 16 profiles generated from an orthogonal fractional factorial design. Although this design reduced the number of profiles from 64 possible combinations and helped manage respondent burden, ranking fatigue was not formally assessed. In addition, the analysis focused on main effects and did not estimate interaction effects among attributes. Holdout profiles were not included, and the stability of individual-level utility estimates was not separately examined. Therefore, Pearson’s R should be interpreted only as an indicator of internal correspondence between observed and estimated rankings within the conjoint task, rather than as evidence of predictive validity or behavioral realism. Future studies could include holdout profiles, examine interaction effects, and assess the stability of individual-level utility estimates to strengthen the validation of conjoint results. In addition, the cluster analysis should be interpreted as exploratory. The four-cluster solution was selected based on interpretability and distinctiveness of constraint-weighting patterns, but objective validation procedures such as silhouette statistics, the elbow method, information criteria, or replication with an independent sample were not conducted. Therefore, the cluster labels should be understood as descriptive summaries rather than empirically verified behavioral types or developmental trajectories.

Fourth, the segmentation analysis was primarily based on participants’ cognitive weighting patterns across constraint attributes. While this approach effectively captures differences in perceived importance, it does not fully account for other psychological and behavioral variables that may shape constraint perception, such as motivation, self-efficacy, and goal orientation. Moreover, the underlying mechanisms through which these variables influence constraint evaluation were not directly examined. Future studies could extend this work by integrating such constructs into the segmentation framework or by employing advanced analytical techniques, such as latent class analysis or structural equation modeling, to provide a more comprehensive understanding of the relationships among psychological factors and constraint evaluation.

Fifth, in this study, respondents were classified into high and low involvement groups using the sample mean as a cut-off value. Although this approach was useful for exploratory group-based comparison in the conjoint analysis, it may have reduced variability in the continuous involvement construct and may have introduced the possibility of misclassification among respondents whose scores were close to the mean. Therefore, the involvement-based comparison should be interpreted as an exploratory contrast between relatively higher and lower involvement groups rather than as evidence of clearly distinct psychological categories.

Sixth, the interpretation of cognitive weighting should be approached cautiously. Although conjoint analysis estimates relative importance and part-worth utility values, these estimates are derived from respondents’ rankings of hypothetical profiles and do not directly observe underlying cognitive mechanisms. Therefore, the findings should be interpreted as task-based perceived constraint prioritization rather than as definitive evidence of stable psychological weighting processes. Future studies could combine conjoint analysis with qualitative interviews, experimental designs, or longitudinal behavioral data to examine whether these inferred weighting structures correspond to actual cognitive processes and exercise behavior.

### 5.3. Future Study Directions

Building on the limitations identified in this study, several directions for future research can be proposed.

First, future studies should include more diverse samples, including non-participants, physically inactive individuals, and individuals who have discontinued exercise. Because the present study focused only on adults with recent exercise participation experience, future research should compare constraint weighting patterns between active exercisers and non-participants. Such comparisons would make it possible to determine whether the perceived constraints identified in this study are specific to recent exercisers or whether they also apply to individuals who do not currently participate in exercise.

Second, future research should collect more detailed sample characteristics that may influence exercise participation constraints. In particular, future studies should include variables such as physical health status, mental health status, diagnosed diseases or pathologies, living area, facility accessibility, income, employment status, and family or work-related responsibilities. These variables may shape how individuals perceive fatigue, time burden, cost burden, and environmental constraints. Incorporating such factors would allow researchers to provide a more comprehensive explanation of differences in perceived constraint structures across individuals and groups.

Third, future studies could include holdout profiles, examine interaction effects, assess the stability of individual-level utility estimates, and validate participant segmentation using objective cluster validation procedures to strengthen the robustness of conjoint and segmentation results.

Fourth, future research should incorporate key psychological variables, such as motivation, self-efficacy, and goal orientation, into the analytical framework. While this study focused on cognitive weighting patterns across constraint attributes, it did not directly examine the psychological processes underlying these evaluations. Integrating such variables would allow for a more comprehensive understanding of how individuals perceive, interpret, and prioritize participation constraints, thereby extending the theoretical contribution of constraint-based research in exercise behavior.

Fifth, future research should also consider alternative approaches to examining the role of exercise involvement. Rather than dichotomizing involvement into high and low groups, future studies could treat involvement as a continuous moderating variable or apply latent profile analysis to identify naturally occurring involvement profiles. These approaches may preserve more information from the original scale and provide a more nuanced understanding of how involvement shapes the perceived prioritization of exercise participation constraints.

## Figures and Tables

**Table 1:** Respondents’ demographic characteristics.

Variable	Category	n	%
Gender	Male	162	57.2
	Female	121	42.8
Age	20s	64	22.6
	30s	89	31.4
	40s	78	27.6
	50 years or older	52	18.4
Weekly exercise frequency	1~2 times per week	107	37.8
	3–4 times per week	131	46.3
	Everyday	45	15.9
Duration of exercise participation	Less than 6 months	54	19.1
	6 months to less than 1 year	98	34.6
	1 to less than 3 years	80	28.3
	3 years or more	51	18.0

**Table 5:** Conjoint profiles of exercise participation constraints.

Profiles	Interest in Exercise	Physical Fatigue	Partner Availability	Social Norm Pressure	Cost Burden	Time Burden
1	Low interest	Easily tired	Available	High pressure	Low cost	Sufficient time
2	High interest	Rarely tired	Not available	Low pressure	High cost	Insufficient time
3	High interest	Easily tired	Not available	Low pressure	Low cost	Sufficient time
4	Low interest	Rarely tired	Available	Low pressure	High cost	Sufficient time
5	High interest	Rarely tired	Available	High pressure	Low cost	Insufficient time
6	Low interest	Rarely tired	Not available	High pressure	Low cost	Sufficient time
7	High interest	Easily tired	Available	High pressure	High cost	Sufficient time
8	Low interest	Rarely tired	Not available	Low pressure	Low cost	Insufficient time
9	High interest	Rarely tired	Available	Low pressure	Low cost	Sufficient time
10	Low interest	Easily tired	Not available	High pressure	High cost	Insufficient time
11	High interest	Rarely tired	Not available	High pressure	High cost	Sufficient time
12	Low interest	Easily tired	Available	Low pressure	Low cost	Insufficient time
13	High interest	Easily tired	Not available	High pressure	Low cost	Insufficient time
14	Low interest	Rarely tired	Available	High pressure	High cost	Insufficient time
15	Low interest	Easily tired	Not available	Low pressure	High cost	Sufficient time
16	High interest	Easily tired	Available	Low pressure	High cost	Insufficient time

**Table 6:** Exploratory factor analysis and reliability analysis of exercise involvement.

Construct	Scale Items	Factor Loading
Exercise involvement	Exercise is important to me.	0.865
	2. Exercise is a valuable activity to me.	0.847
	3. Exercise is necessary for me.	0.844
	4. Exercise provides me with fun and interest.	0.806
	5. Exercise is an activity that is related to my daily life.	0.786
	6. Exercise offers something of special interest to me.	0.743
	Eigenvalue	4.083
	Variance (%)	68.055
	Cumulative variance (%)	68.055
	Cronbach α	0.859
	KMO measure of sampling adequacy = 0.864 Bartlett’s test of sphericity =742.318, *df* =15, sig = 0.000	

**Table 7:** Classification of exercise involvement.

Construct	Classification	Involvement Value (Mean)	Number of Participants (%)
Exercise involvement	High exercise involvement	4.29 (3.84)	176 (62.2)
	Low exercise involvement	3.22 (3.84)	107 (37.8)

**Table 8:** Relative importance of attributes of exercise participation constraints for all participants.

Constraints Dimension	Constraints’ Attributes	Constraints’ Level	PWU	RI (%)	Rating
Intrapersonal constraints	Interest in exercise	High interest	0.842	26.412	1
		Low interest	−0.842		
	Physical fatigue	Rarely tired	0.615	20.103	2
		Easily tired	−0.615		
Interpersonal constraints	Partner availability	Available	0.294	12.004	5
		Not available	−0.294		
	Social norm pressure	Low pressure	0.158	11.010	6
		High pressure	−0.158		
Structural constraints	Cost burden	Low cost	0.341	13.187	4
		High cost	−0.341		
	Time burden	Sufficient time	0.502	17.284	3
		Insufficient time	−0.502		
Pearson’s R = 0.871 (*p* < 0.001)					
PWU = Part-worth utility; RI = Relative importance					

**Table 9:** Relative importance of attributes of exercise participation constraints for highly exercise-involved participants.

Constraints Dimension	Constraints’ Attributes	Constraints’ Level	PWU	RI (%)	Rating
Intrapersonal constraints	Interest in exercise	High interest	0.611	19.236	2
		Low interest	−0.611		
	Physical fatigue	Rarely tired	0.284	14.358	4
		Easily tired	−0.284		
Interpersonal constraints	Partner availability	Available	0.192	11.127	5
		Not available	−0.192		
	Social norm pressure	Low pressure	0.134	9.245	6
		High pressure	−0.134		
Structural constraints	Cost burden	Low cost	0.416	17.842	3
		High cost	−0.416		
	Time burden	Sufficient time	0.873	28.192	1
		Insufficient time	−0.873		
Pearson’s R = 0.855 (*p* < 0.001)					
PWU = Part-worth utility; RI = Relative importance					

**Table 10:** Relative importance of attributes of exercise participation constraints for low-exercise-involved participants.

Constraints Dimension	Constraints’ Attributes	Constraints’ Level	PWU	RI (%)	Rating
Intrapersonal constraints	Interest in exercise	High interest	0.912	29.384	1
		Low interest	−0.912		
	Physical fatigue	Rarely tired	0.421	14.173	4
		Easily tired	−0.421		
Interpersonal constraints	Partner availability	Available	0.248	11.624	5
		Not available	−0.248		
	Social norm pressure	Low pressure	0.152	9.002	6
		High pressure	−0.152		
Structural constraints	Cost burden	Low cost	0.563	18.917	2
		High cost	−0.563		
	Time burden	Sufficient time	0.472	16.900	3
		Insufficient time	−0.472		
Pearson’s R = 0.846 (*p* < 0.001)					
PWU = Part-worth utility; RI = Relative importance					

**Table 11:** Cluster analysis of exercise participation constraints.

Constraints’ Attributes	Cluster 1 (n = 72)	Cluster 2 (n = 65)	Cluster 3 (n = 77)	Cluster 4 (n = 69)	F
Interest in exercise	34.218	19.326	21.145	18.472	56.381 ***
Physical fatigue	18.412	29.784	19.217	16.308	47.952 ***
Partner availability	12.308	13.241	10.562	11.184	3.218 *
Social norm pressure	8.443	8.484	2.581	7.212	1.842
Cost burden	11.482	12.641	14.853	28.507	44.289 ***
Time burden	15.137	16.524	31.642	18.317	53.164 ***

Note: *** *p* < 0.001, * *p* < 0.05.

**Table 12:** Results of the chi-square test.

Variable	Category	Cluster 1 (n = 72)	Cluster 2 (n = 65)	Cluster 3 (n = 77)	Cluster 4 (n = 69)	χ^2^	*p*-Value
Gender	Male	54	40	51	47	2.960	0.398
	Female	18	25	26	22		
Age	20s	**24**	14	16	10	19.600	0.021 *
	30s	20	18	**30**	21		
	40s	17	**23**	22	16		
	50 years or older	11	10	9	**22**		
Weekly exercise frequency	1~2 times per week	**40**	25	23	19	30.291	0.001 **
	3–4 times per week	26	**30**	**31**	**44**		
	Everyday	6	10	23	6		
Duration of exercise participation	Less than 6 months	20	14	8	12	24.686	0.003 **
	6 months to less than 1 year	**28**	**26**	22	**22**		
	1 to less than 3 years	18	20	**26**	16		
	3 years or more	6	5	21	19		

Note: Bold values represent the maximum number of participants in each cluster. ** *p* < 0.01; * *p* < 0.05.

**Table 13:** Cluster classification.

Cluster	Cluster Name	Demographic Profile (Age, Weekly Exercise Frequency, Duration of Exercise Participation)	Key Characteristics
Cluster 1	Interest-constrained beginners	20s/1–2 times per week/ 6 months to less than 1 year	Participants with low exercise frequency and short participation experience, primarily constrained by low interest in exercise
Cluster 2	Fatigue-sensitive participants	40s/3–4 times per week/ 6 months to less than 3 years	Participants with moderate exercise frequency and experience, whose participation is mainly limited by physical fatigue
Cluster 3	Time-constrained active participants	30s/daily participation/ 3 years or more	Highly active participants with long-term experience who frequently exercise but perceive significant time constraints
Cluster 4	Cost-sensitive experienced participants	50 years or older/3–4 times per week/3 years or more	Experienced participants with a stable exercise habit whose participation is primarily influenced by cost burden

## Data Availability

The data presented in this study are available on request from the corresponding author.
